# Targeting adrenergic receptors to mitigate invariant natural killer T cells-induced acute liver injury

**DOI:** 10.1016/j.isci.2023.107947

**Published:** 2023-09-16

**Authors:** Michelangelo Bauwelz Gonzatti, Beatriz Marton Freire, Maísa Mota Antunes, Gustavo Batista de Menezes, Jhimmy Talbot, Jean Pierre Schatzmann Peron, Alexandre Salgado Basso, Alexandre Castro Keller

**Affiliations:** 1 Department of Microbiology, Immunology and Parasitology, Federal University of São Paulo (UNIFESP/EPM), Rua Botucatu, 862, 4th floor, São Paulo 04023-062, Brazil; 2 Department of Morphology, Federal University of Minas Gerais, Av. Antônio Carlos, 6627, Minas Gerais 31270-910, Brazil; 3 Fred Hutchinson Cancer Center, 1100 Fairview Avenue N, Seattle, WA 98109-1024, USA; 4 Department of Immunology-ICB IV, University of São Paulo, Av. Prof. Lineu Prestes, 1730, São Paulo 05508-900, Brazil

**Keywords:** Cancer, Cell biology, Components of the immune system, Immunology

## Abstract

Invariant Natural Killer T (iNKT) cell activation by α-galactosylceramide (αGC) potentiates cytotoxic immune responses against tumors. However, αGC-induced liver injury is a limiting factor for iNKT-based immunotherapy. Although adrenergic receptor stimulation is an important immunosuppressive signal that curbs tissue damage induced by inflammation, its effect on the antitumor activity of invariant Natural Killer T (iNKT) cells remains unclear. We use mouse models and pharmacological tools to show that the stimulation of the sympathetic nervous system (SNS) inhibits αGC-induced liver injury without impairing iNKT cells’ antitumoral functions. Mechanistically, SNS stimulation prevents the collateral effect of TNF-α production by iNKT cells and neutrophil accumulation in hepatic parenchyma. Our results suggest that the modulation of the adrenergic signaling can be a complementary approach to αGC-based immunotherapy to mitigate iNKT-induced liver injury without compromising its antitumoral activity.

## Highlights

• iNKT cells are resistant to the suppressive effects of adrenergic signaling
• Adrenergic signaling mitigates iNKT-induced liver injury
• Sympathetic nervous system pharmacologic modulation mitigates iNKT-induced liver injury

## Graphical abstract

**Figure:**
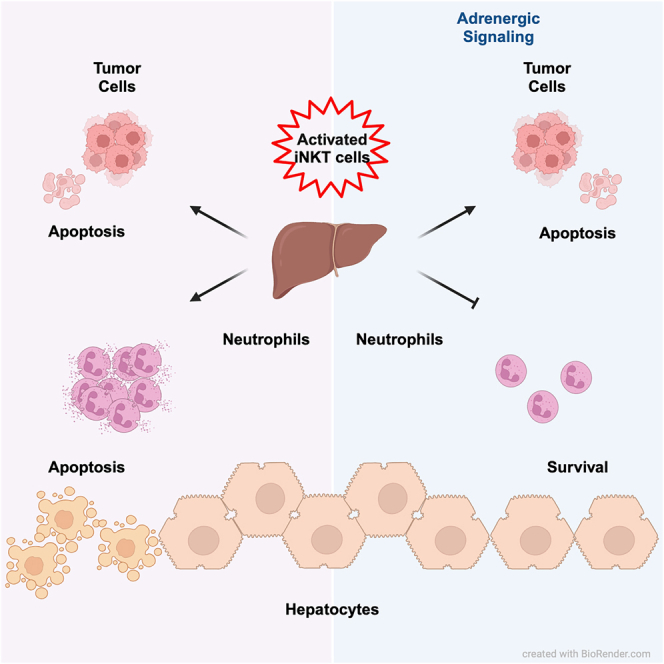

## Introduction

Despite medical advancements in the last decades, cancer remains one of the most significant challenges in clinical practices, especially in cases of advanced or recurrent disease, where treatment options are limited. In this sense, the stimulation of invariant Natural Killer T (iNKT) cells by specific agonists, such as α-galactosylceramide (αGC), appeared as an alternative to cancer management.^1^^,^^2^^,^^3^^,^^4^

iNKT cells, also known as NKT/NKT-I, are a CD1d-restricted population of non-conventional T lymphocytes with potent modulatory capacity and selectivity for glycolipids, properties shared by human, primate, and non-primate species.^5^ Indeed, murine experimental models were the first to describe the existence of an invariant T lymphocyte sharing distinct characteristics with T lymphocytes and NK cells, with strong antitumor ability.^1^^,^^6^ After this discovery, experimental models have been extensively used to improve the knowledge of iNKT cells’ biology, its agonists, and, thereby, iNKT-based immunotherapy.^7^^,^^8^

In brief, following αGC-stimulation, iNKT cells exert direct cytotoxic activity against CD1d-bearing tumors and can transactivate NK cells and potentiate the effector activity of specific antitumor T cells, especially the CD8^+^ T cell subset.^9^^,^^10^^,^^11^ Because the efficacy of the antimetastatic effect of αGC treatment depends on the sequential activation of DCs, NK cells, and CD8 T lymphocytes triggered by IFN-γ-producing iNKT cells, especially against CD1d-negative tumors, any external events compromising this cascade of immune reactions may impair αGC immunotherapy.^12^^,^^13^

A growing body of evidence supports the idea that the sympathetic nervous system (SNS), through the production of norepinephrine (NE) and primarily via β_2_-adrenergic receptor (β_2_AR) signaling, can act as an immunomodulatory neuronal system. NE impairs the cytotoxic activity of both NK cells and CD8^+^ T lymphocytes, favors the polarization of CD4^+^ T lymphocytes toward a Th2 profile, and modulates innate immunity to restrain tissue damage due to uncontrolled inflammatory responses.^14^^,^^15^^,^^16^^,^^17^^,^^18^^,^^19^^,^^20^

Although naive Th0 CD4^+^ are bipotential and Th1 and Th2 responses are mutually exclusive, NE-induced Th2 polarization seems to be mechanistically related to the signaling via β_2_AR, whose expression is sustained under Th1 but not Th2 driving conditions.^21^^,^^22^ In contrast to conventional T lymphocytes, thymus-emigrated iNKT cells exhibit a memory/effector profile and express genes related to different adrenergic receptors, including the *Adrb2*, which encodes β_2_AR.^23^^,^^24^ Therefore, the expression of ARs in iNKT cells does not seem to be regulated in the same fashion as in conventional T lymphocytes, and their impact on iNKT cells’ biology remains to be clarified.

Considering the potent immunomodulatory ability of iNKT cells and their importance as a target for immunotherapy, it is essential to determine the influence of adrenergic signaling on iNKT cell activity and antitumoral functions.^8^

For this purpose, we used mice genetically deficient for α_2A_ and α_2C_AR (*Adra2ac*^−/−^) as a genetic model of hyperactivation of the SNS.^25^^,^^26^ These receptors promote a negative feedback mechanism controlling catecholamine secretion, and due to their absence, *Adra2ac*^−/−^ mice exhibit an NE hypersecretion phenotype.^25^ Using this model and pharmacologic hyperactivation of the SNS, we found that adrenergic stimulation prevents liver damage induced by iNKT cell activation without affecting their antitumoral activity.

## Results

### Invariant Natural Killer T cells are refractory to adrenergic signaling

**Figure 1:**
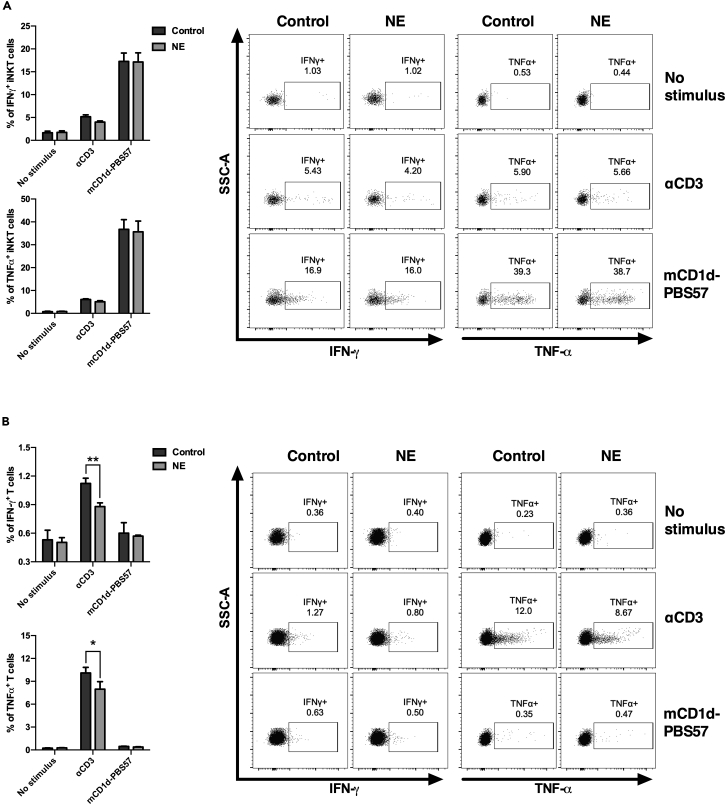
Noradrenergic signaling does not inhibit the *in vitro* activation of iNKT cells (A and B) Splenic cells from C57BL/6J mice were pre-incubated with NE and then stimulated with anti-CD3 (αCD3) or mCD1d/PBS57 in the presence of anti-CD28. The production of IFN-γ and TNF-α were evaluated by flow cytometry. Graphs represent the percentage of cytokine-producing iNKT cells (CD3^+^mCD1d/PBS57^+^) (A) or conventional T cells (CD3^+^mCD1d/PBS57^−^NK1.1^-^) (B). Data represent the mean ± SEM of 2 independent experiments (n = 5). ∗∗p < 0.01; ∗p < 0.05.

Although previous data showed that iNKT cells express mRNAs encoding α and β-adrenergic receptors, the direct effect of NE signaling on their biological activity remains unclear.^24^ Therefore, to determine the impact of adrenergic signaling on iNKT cell activity, we first incubated naive splenic cells from C57BL/6J WT mice with an anti-CD3 antibody or with PBS57, an αGC analog loaded in CD1d monomers in the presence or absence of NE (Figure 1). While the maximum iNKT cell response was achieved using the cognate antigen, adrenergic stimulation did not influence the production of IFN-γ or TNF-α following stimulation with anti-CD3 or PBS-57 (Figure 1A). In contrast, conventional T lymphocytes exhibited a lower frequency of IFN-γ and TNF-α-producing T cells under NE stimulation compared to control samples (Figure 1B). These results are in concordance with previous reports describing that NE signaling impairs the acquisition of a Th1 effector profile by naive CD3^+^ T lymphocytes upon anti-CD3 stimulation.^18^ Therefore, iNKT cells, but not conventional T lymphocytes, are refractory to the inhibitory effect of adrenergic signaling.

**Figure 2:**
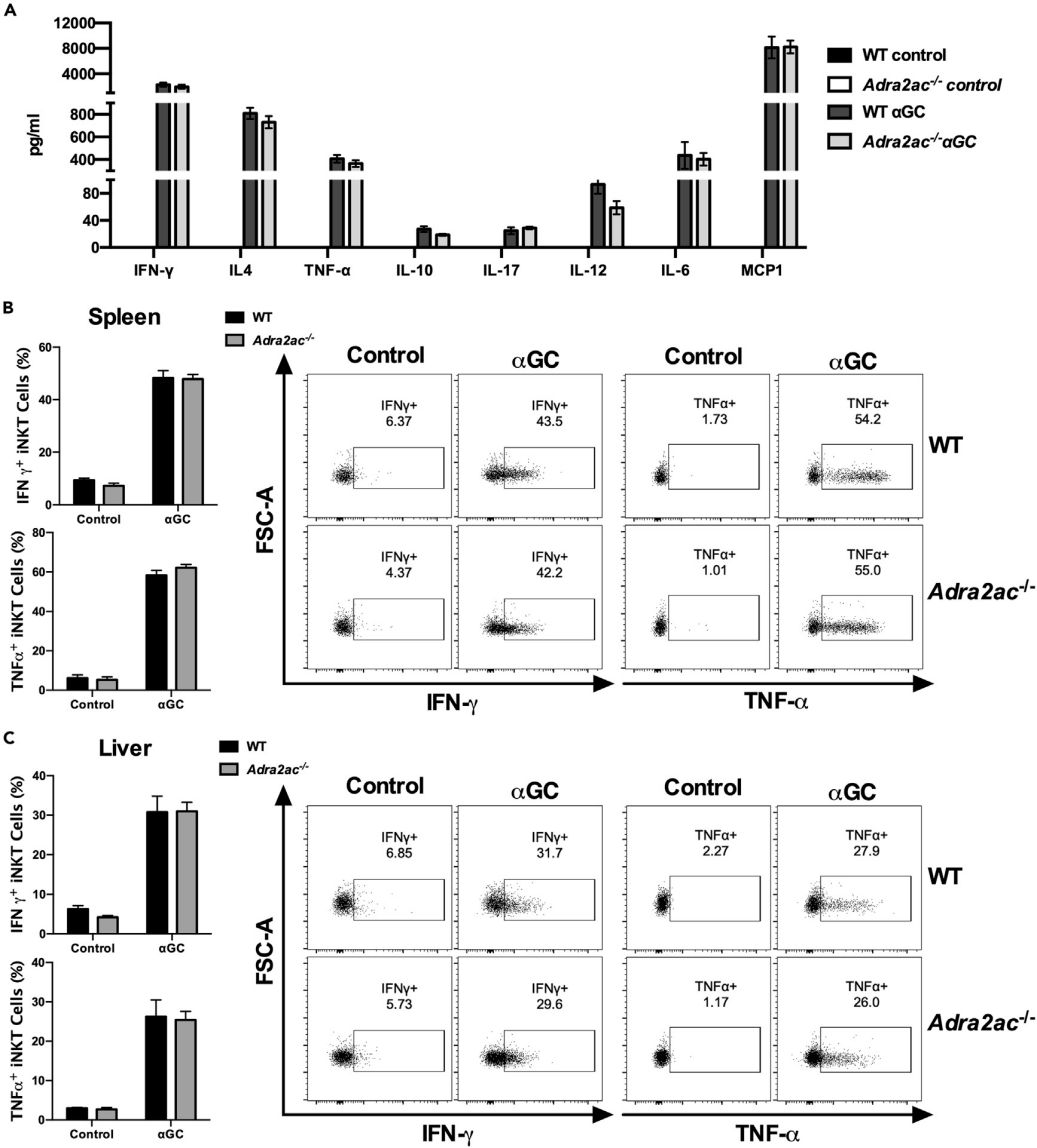
Hyperactivation of the adrenergic system does not inhibit cytokine production following iNKT cell activation *in vivo* (A–C) C57BL/6J WT and *Adra2ac*^−/−^ mice were treated with αGC (2μg/animal), and serum cytokine production was evaluated after 4 h (A). Mean ± SEM of 3 different experiments (n = 4–6), all data had a minimum p < 00.5 versus the respective control group. For the *ex vivo*, intracellular cytokine assay, splenic (B) and hepatic (C) cells were harvested 90 min after αGC administration and incubated with brefeldin and monensin for 2 h. Subsequently, staining was performed for the detection of IFN-γ and TNF-α production by iNKT cells. Data represent the mean ± SEM of 2 independent experiments (n = 5), p < 0.0001 versus the respective control group.

Next, we took advantage of the *Adra2ac*^−/−^ mouse, a prototypical model of NE hypersecretion, to study *in vivo* the influence of adrenergic signaling on the iNKT cells’ response to αGC.^26^ αGC injection increases systemic levels of several immunomodulatory molecules, such as IL-6, IFN-γ, TNF-α, IL-4, IL-10, IL-17, IL-12 and MCP-1, without a significant difference between WT and *Adra2ac*^−/−^ animals (Figure 2A). Flow cytometric analysis indeed revealed that the production of cytokines by splenic and hepatic iNKT cells following αGC injection was similar in both mouse strains, with rapid production of IFN-γ and TNF-α upon TCR engagement (Figures 2B and 2C).

Notably, no difference in the frequency of thymic or peripheral iNKT cells was observed between the C57BL/6J WT and *Adra2ac*^−/−^ mouse strain (Figure S2). Therefore, these results support the hypothesis that adrenergic signaling does not influence the iNKT cell response to αGC and, consequently, the cascade of events that follows its stimulation.

### Adrenergic signaling inhibits α-galactosylceramide-induced liver injury

**Figure 3:**
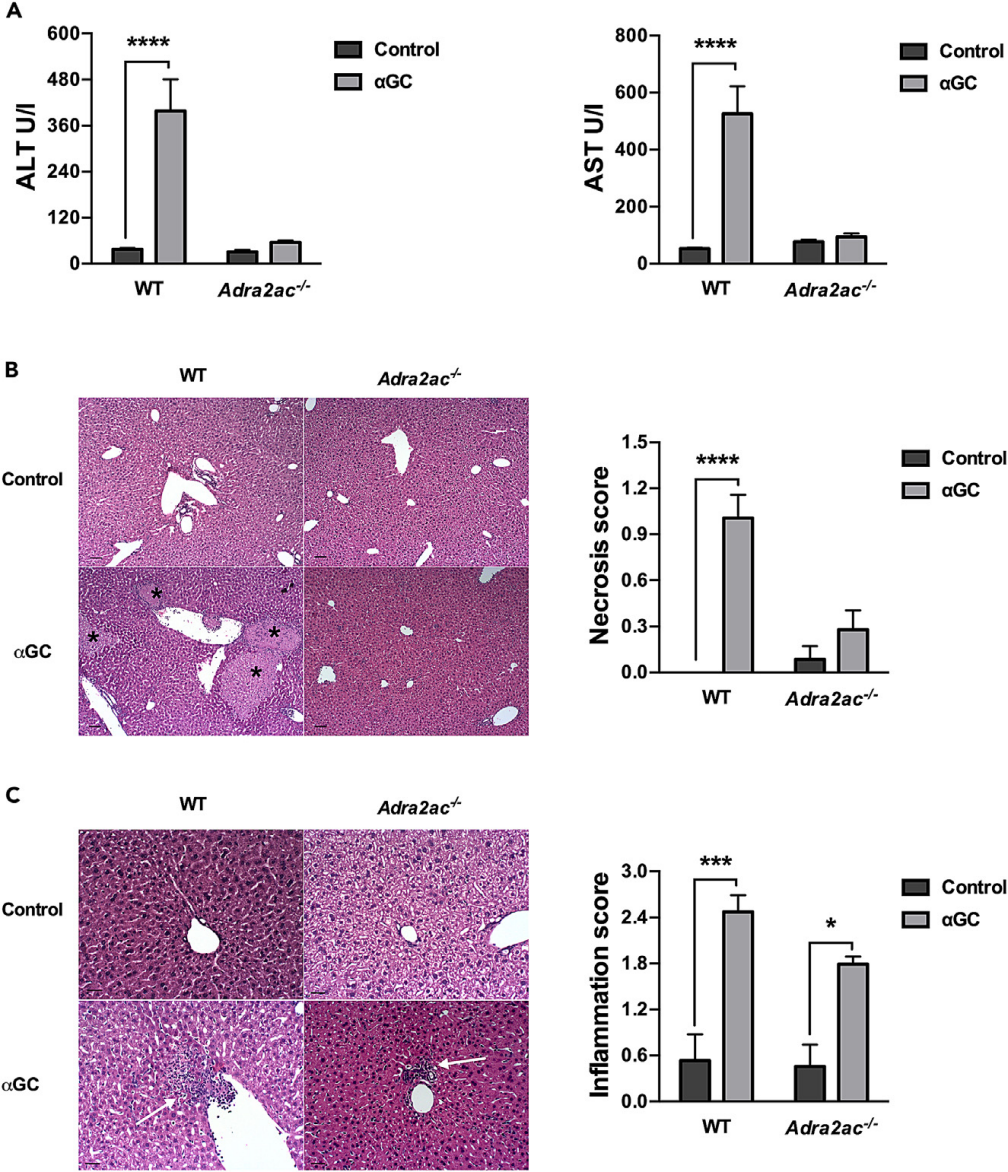
Adrenergic signaling mitigates αGC-induced liver injury (A–C) C57BL/6J WT and *Adra2ac*^−/−^ mice were treated with αGC (2μg/animal). Serum levels of ALT and AST (A) were evaluated after 16 h, and liver tissue was collected for histological analysis. Representative images and graphs for necrosis (B) and inflammation (C) scores are presented. Black asterisks indicate necrotic areas, and white arrows indicate areas of cellular infiltration indicative of inflammation. Data represent the mean ± SEM of 2 independent experiments (n = 5). ∗∗∗∗p < 0.0001; ∗∗∗p < 0.001; ∗p < 0.05. scale bar 100μm (B) or 50μm (C).

The production of pro-inflammatory cytokines, especially TNF-α, in response to αGC has been associated with liver injury, characterized by increased systemic levels of hepatic enzymes (ALT and AST) and alterations in the hepatic parenchyma, such as the appearance of necrotic areas.^27^ Indeed, WT animals treated with αGC exhibited a significant increase in systemic levels of ALT and AST associated with signs of hepatic injury, including necrotic areas and robust inflammatory foci (Figures 3A–3C). In contrast, the *Adra2ac*^−/−^ animals treated with αGC did not present liver dysfunction, exhibiting systemic levels of ALT and AST comparable to those of control, non-treated animals (Figure 3A). Moreover, histological analysis revealed the preservation of the hepatic tissue in the *Adra2ac*^−/−^ animals despite a significant influx of inflammatory cells into the liver tissue (Figures 3B and 3C). Therefore, these results demonstrate that although adrenergic signaling did not inhibit the iNKT cells’ activity and inflammatory responses triggered by αGC stimulation, it provides a hepatoprotective effect against iNKT-induced tissue damage.

### Adrenergic signaling does not inhibit the control of melanoma growth by α-galactosylceramide immunotherapy

**Figure 4:**
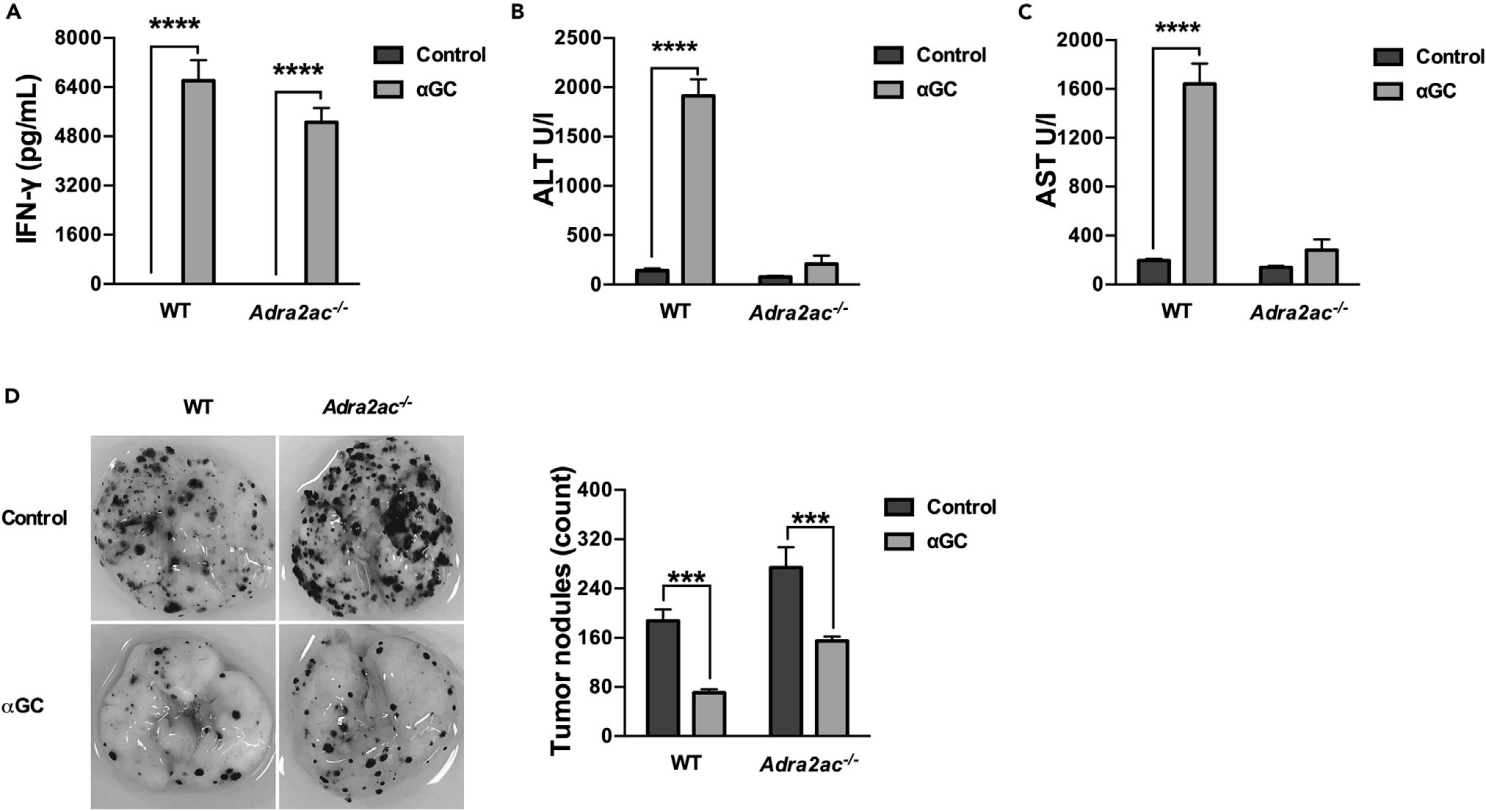
Adrenergic signaling does not influence the efficiency of αGC immunotherapy against melanoma cells (A–D) C57BL/6J WT and *Adra2ac*^−/−^ mice were grafted with B16F10 cells and subsequently treated with αGC (2μg/animal). Serum levels of IFN-γ (A), ALT (B) and AST (C) were evaluated after 16 h. Pulmonary metastatic nodules were quantified after 15 days (D). Data represent the mean ± SEM of 2 different experiments (n = 5). ∗∗∗∗p < 0.0001; ∗∗∗p < 0.001.

Previous studies reported that β_2-_adrenergic receptor signaling undermines the immunosurveillance against tumor cells by impairing the activity of NK cells and CD8^+^ T lymphocytes.^16^^,^^28^ In concordance with these findings, *Adra2ac*^−/−^ mice are more susceptible to B16F10 lung metastasis than their WT counterparts (∗p < 0.05; Figure 4D). Therefore, considering that the effectiveness of αGC immunotherapy relies on a cascade of events triggered by the production of IFN-γ by iNKT cells, it was essential to determine the impact of NE hypersecretion on the antitumor efficacy of αGC treatment.^12^

The αGC-treated WT group exhibited a substantial increase in systemic IFN-γ levels, hepatic enzymes (ALT and AST), and a significant reduction in the number of lung nodules in comparison to the non-treated group (Figures 4A–4D), reinforcing the notion that in addition to controlling tumor growth, αGC immunotherapy also results in liver injury. The tumor-bearing *Adra2ac*^−/−^ mice responded positively to αGC injection, exhibiting a significant increase in systemic IFN-γ and control of tumor growth without any significant alteration in the systemic levels of hepatic enzymes, thus corroborating the hepatoprotective effect of adrenergic signaling (Figures 4A–4D). Therefore, while the sympathetic nervous system activation might impair natural immunosurveillance against tumors, the iNKT-mediated immune response to melanoma cells is refractory to its immunosuppressive effects.

### Pharmacological stimulation of the sympathetic nervous system inhibits α-galactosylceramide-induced liver injury

**Figure 5:**
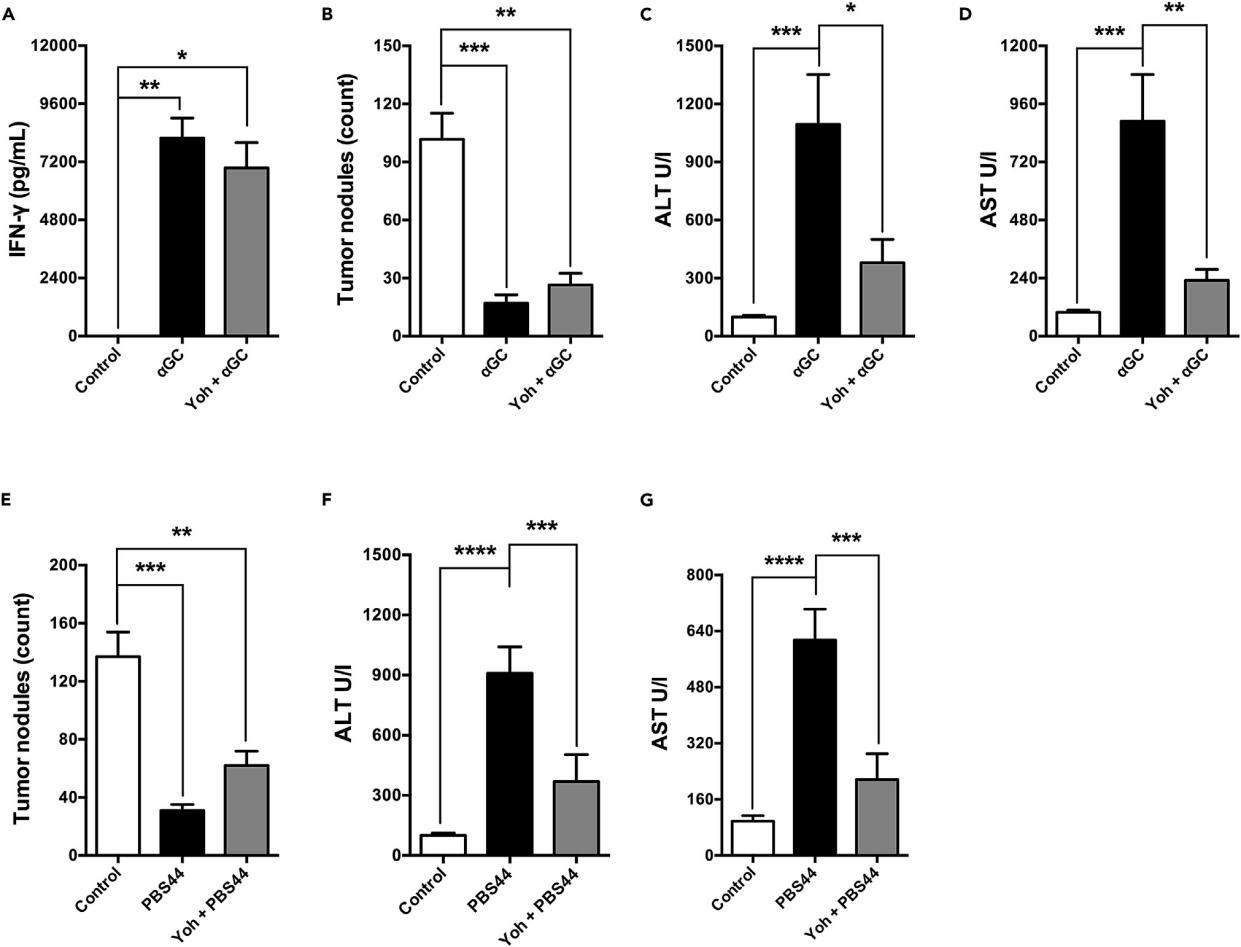
Pharmacological stimulation of the sympathetic nervous system by yohimbine controls hepatic damage induced by iNKT activation without impairing its antitumoral activity (A–D) C57BL/6J WT mice were treated with Yoh prior to grafting with B16F10 cells and subsequent αGC treatment (2μg/animal). Serum levels of IFN-γ (A), ALT (C) and AST (D) were evaluated after 16 h. Pulmonary metastatic nodules were quantified after 15 days (B). (E) C57BL/6J WT mice were treated with Yoh prior to grafting and subsequent PBS44 treatment (2μg/animal). Pulmonary metastatic nodules were quantified after 15 days. (F and G) Serum levels of ALT (F) and AST (G) were evaluated after 16 h. Data represent the mean ± SEM of 2 independent experiments (n = 5). ∗∗∗∗p < 0.0001; ∗∗∗p < 0.001; ∗∗p < 0.01; ∗p < 0.05.

Considering the hepatoprotective effect resulting from the hyperactivity of the adrenergic fibers observed in *Adra2ac*^−/−^ mice, we hypothesized that a punctual stimulation of the SNS should also be able to mitigate liver damage without affecting the αGC antimetastatic ability. To test this idea, WT animals received a single injection of yohimbine (Yoh), an α_2_-adrenergic antagonist that mimics the lack of control of NE secretion due to the absence of the α_2A/C_AR, before αGC treatment.^29^^,^^30^ Figure 5 shows that administering Yoh before αGC treatment did not affect IFN-γ production or the antitumor effect associated with iNKT cell activation, compared to control, non-treated mice (Figures 5A and 5B). Moreover, systemic levels of hepatic enzymes in the Yoh group were comparable to those detected in control mice, in contrast to the αGC group, which exhibited a significant increase in systemic levels of ALT and AST (Figures 5C and 5D). Besides αGC, other specific agonists have been developed to improve iNKT-based immunotherapy. Thus, we used the same approach to study the impact of adrenergic signaling on the antimetastatic effect of PBS44, an αGC analog with antitumor activity.^9^ Despite controlling lung metastasis, PBS44 treatment also increased the levels of ALT and AST, which were controlled by pre-treatment with Yoh (Figures 5E–5G). Therefore, these results support the notion that the pharmacological modulation of sympathetic fiber activity during iNKT cells stimulation improves immunotherapy outcomes.

### Adrenergic signaling mitigates invariant natural killer T-induced liver injury through the α_1_ receptor

As adrenergic signaling did not exert a direct suppressive effect on the immune responses triggered by iNKT cell activation, we hypothesized that it could act directly on hepatocyte survival. Analyzing datasets from two different RNAseq liver libraries, we found that, under steady-state conditions, mouse hepatocytes primarily express the *Adra1b* genes.^31^^,^^32^ However, a recent study demonstrated that hepatic injury increases the expression of β_2_AR, which promotes liver regeneration.^33^ Therefore, we employed two strategies to study the stimulatory pathways driving the hepatoprotective effect provided by adrenergic signaling.

First, we used a triple KO model, *Adra2ac*^−/−^*Adrb2*^−/−^, a mouse strain that exhibits hyperactivity of the sympathetic nervous system along with the absence of the β_2_AR. Similar to what was observed in *Adra2ac*^−/−^ mice, no significant alterations in ALT and AST levels were observed after αGC administration, indicating that in our model, β_2_AR signaling is dispensable for the hepatoprotective effect promoted by increased NE release (Figure S3).

**Figure 6:**
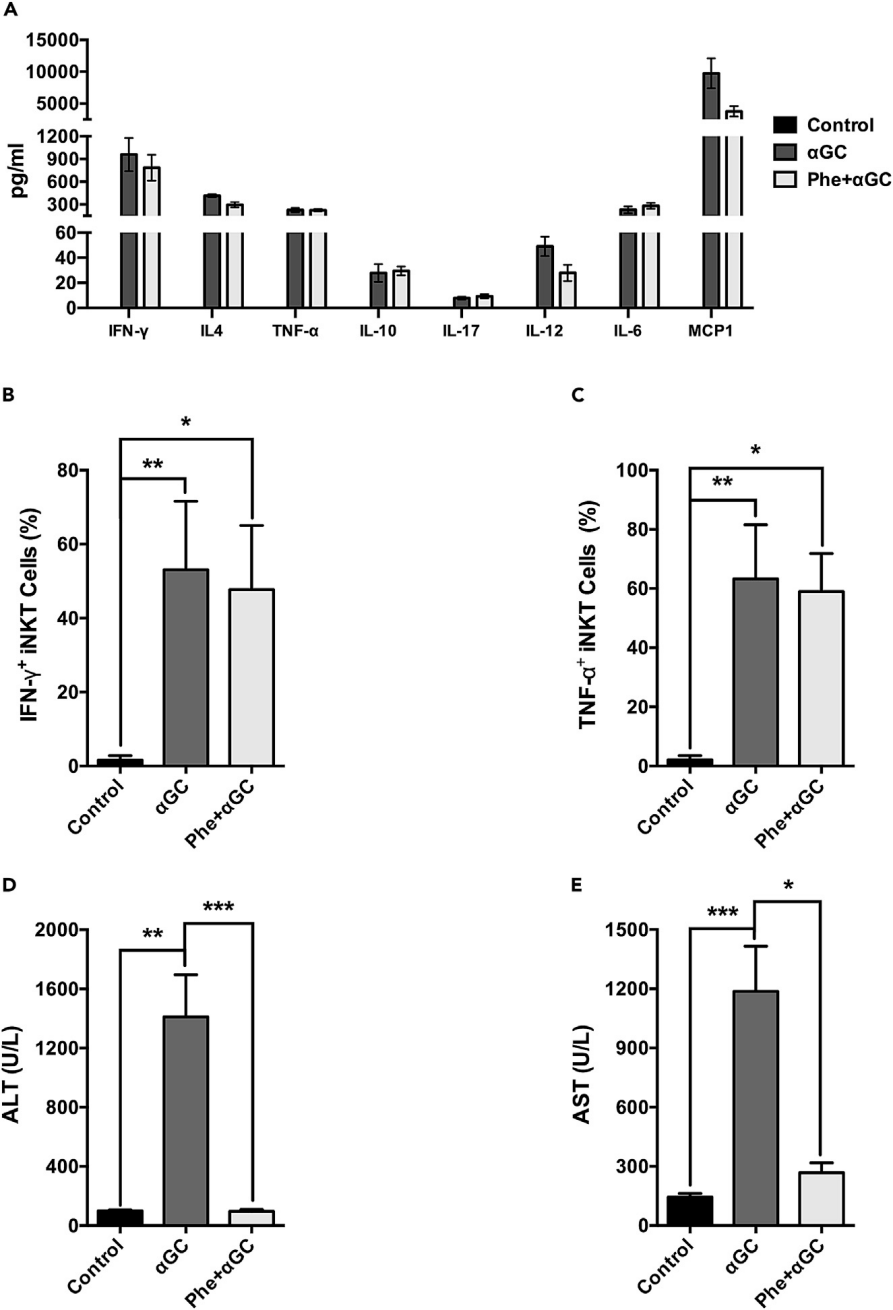
Pharmacological stimulation of α_1_ adrenergic receptor controls hepatic damage induced by activated iNKT cells (A) C57BL/6J WT mice were treated with Phe 30 min prior to αGC treatment (2μg/animal). Serum cytokine production was evaluated after 4 h. (B and C) For the *ex vivo* intracellular cytokine assay, splenic cells were harvested 90 min after αGC administration and incubated with brefeldin and monensin for 2 h, followed by staining for the detection of IFN-γ (B) and TNF-α (C) production by iNKT cells. (D and E) ALT (D) and AST (E) levels in serum were evaluated after 16 h. Data represent the mean ± SD from a single set of two independent experiments (n = 6, B and C); Mean ± SEM of 2 independent experiments, (n = 4–5, A, D, and E). ∗∗∗p < 0.001; ∗∗p < 0.01; ∗p < 0.05.

Next, we pre-treated C57BL/6J WT mice with phenylephrine (Phe), an α_1_AR agonist, before αGC administration.^34^ This approach did not affect the overall development of a systemic pro-inflammatory response or the specific production of IFN-γ or TNF-α by iNKT cells induced by the *in vivo* administration of αGC (Figures 6A–6C). Despite high amounts of pro-inflammatory cytokines, no significant alterations in the ALT and AST levels were observed compared to control mice, in contrast to the αGC-treated group (Figures 6D and 6E).

### Adrenergic signaling fails to increase hepatocyte resistance to apoptosis

**Figure 7:**
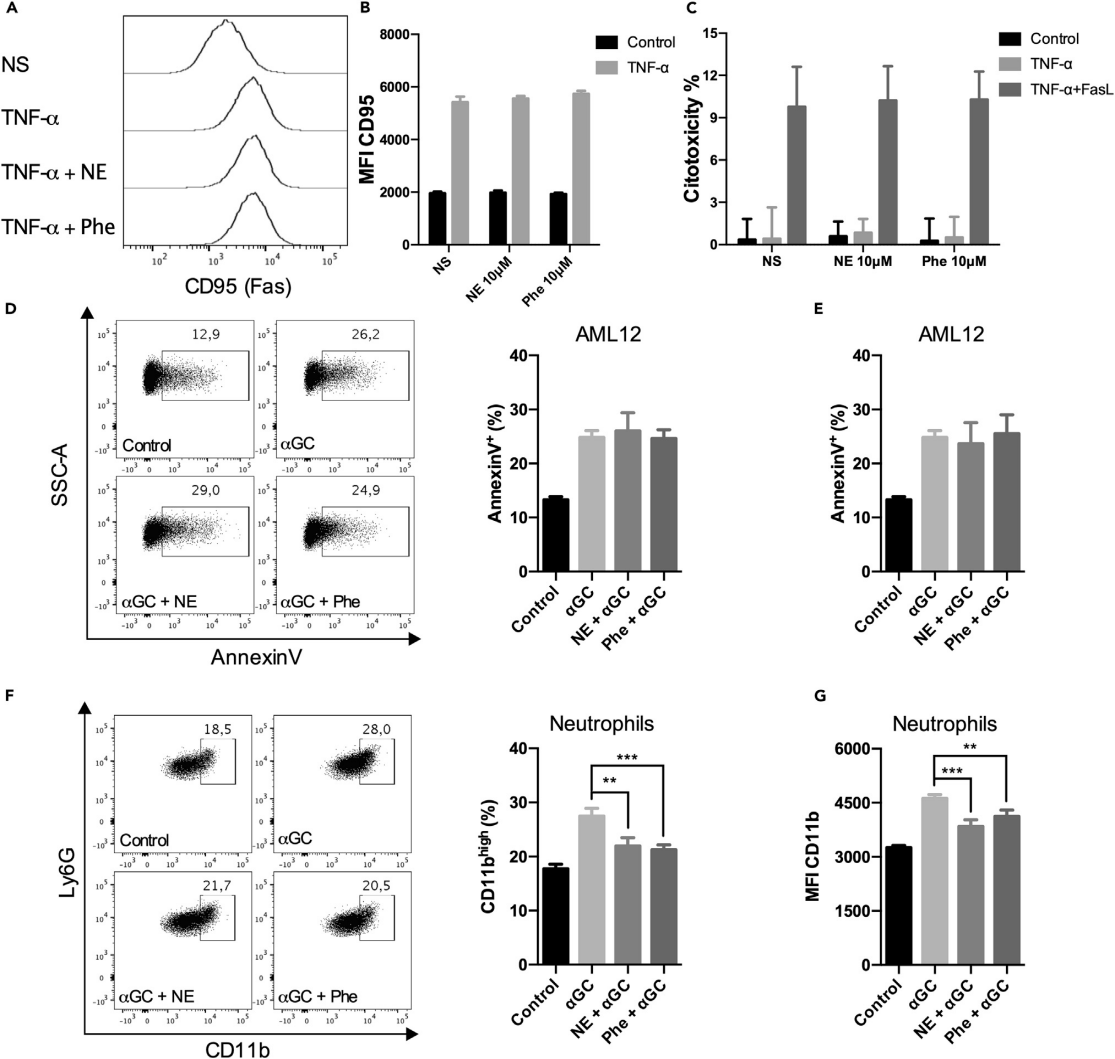
Adrenergic signaling fails to increase hepatocyte resistance to apoptosis (A and B) AML12 cells were pre-treated with NE or Phe for 2 h, treated with TNF-α (20 ng/ml) for 16 h, and the expression of CD95 was evaluated by flow cytometry; graphs represent the median fluorescence intensity (MFI). (C) AML12 cells were pre-treated with NE or Phe for 2 h, stimulated with TNF-α (20 ng/ml) for 4 h, and treated with FasL (100 ng/ml) in the presence of TNF ligand enhancer (1 μg/ml) for additional 12 h; cytotoxicity was evaluated by LDH release in the supernatant, and graphs represent the percentage of cell death. (D) AML12 cells were pulsed with αGC (250 ng/ml), pre-treated with NE or Phe for 2 h and co-cultured with liver leukocytes for 4 h; graphs represent the percentage of apoptotic hepatocytes (SSC/FSC^high^CD45^−^Live/dead^−^AnnexinV^+^). (E–G) Liver leukocytes were pre-treated with NE or Phe for 2 h and co-cultured with AML12 pulsed with αGC for 4 h. Graphs represent the percentage of apoptotic hepatocytes (E), CD11b^high^ neutrophils (SSC/FSC^int^CD45^+^CD11b^+^Ly6G^+^) (F), and CD11b MFI (G). Data represent the mean ± SD from a single set of two independent experiments (n = 5) ∗∗p < 0.01, ∗∗∗p < 0.001.

To determine the effect of adrenergic signaling on hepatocyte survival, we took advantage of the murine-derived hepatocyte cell line, AML12. Although AML12 hepatocytes proved to be insensitive to TNF-α-induced apoptosis, treatment with NE or Phe failed to inhibit the increase in TNF-α-induced Fas expression (Figures 7A and 7B). Next, AML12 were cultured under the same conditions in the presence of FasL and TNF ligand enhancer. Under this condition, activation of the FasL/Fas pathway induced hepatocyte death, which was not inhibited by adrenergic signaling (Figure 7C). Hence, adrenergic signaling does not enhance the hepatocyte resistance to TNF-α/FasL-Fas-induced cell death.

We then co-cultured αGC-pulsed AML12 cells with hepatic leukocytes (HL) for 4 h under two conditions: 1) AML12 cells were pre-treated with NE or Phe for 2 h and subsequently co-cultured with HL; 2) AML12 cells were incubated with HL previously treated with NE or Phe for 2 h.

Pre-treating AML12 cells with NE or Phe did not protect the hepatocytes from αGC-induced apoptosis (AnnexinV^+^) (Figure 7D). Similarly, pre-incubating HL with NE or Phe did not inhibit AML12 apoptosis, thus supporting the idea that adrenergic signaling is ineffective in inhibiting iNKT-induced hepatocyte apoptosis (Figure 7E). In addition to the direct cytotoxic effect on CD1d-expressing target cells, αGC-induced iNKT cell activation has also been associated with the early appearance of pro-inflammatory neutrophils, which contribute to the extension of liver damage.^35^ In this regard, flow cytometric analysis of HL revealed that pre-treatment with NE or Phe impaired the shift of neutrophils toward a more inflammatory profile (Figures 7F and 7G).

### Adrenergic signaling inhibits α-galactosylceramide-induced migration of inflammatory cells to hepatic parenchyma

**Figure 8:**
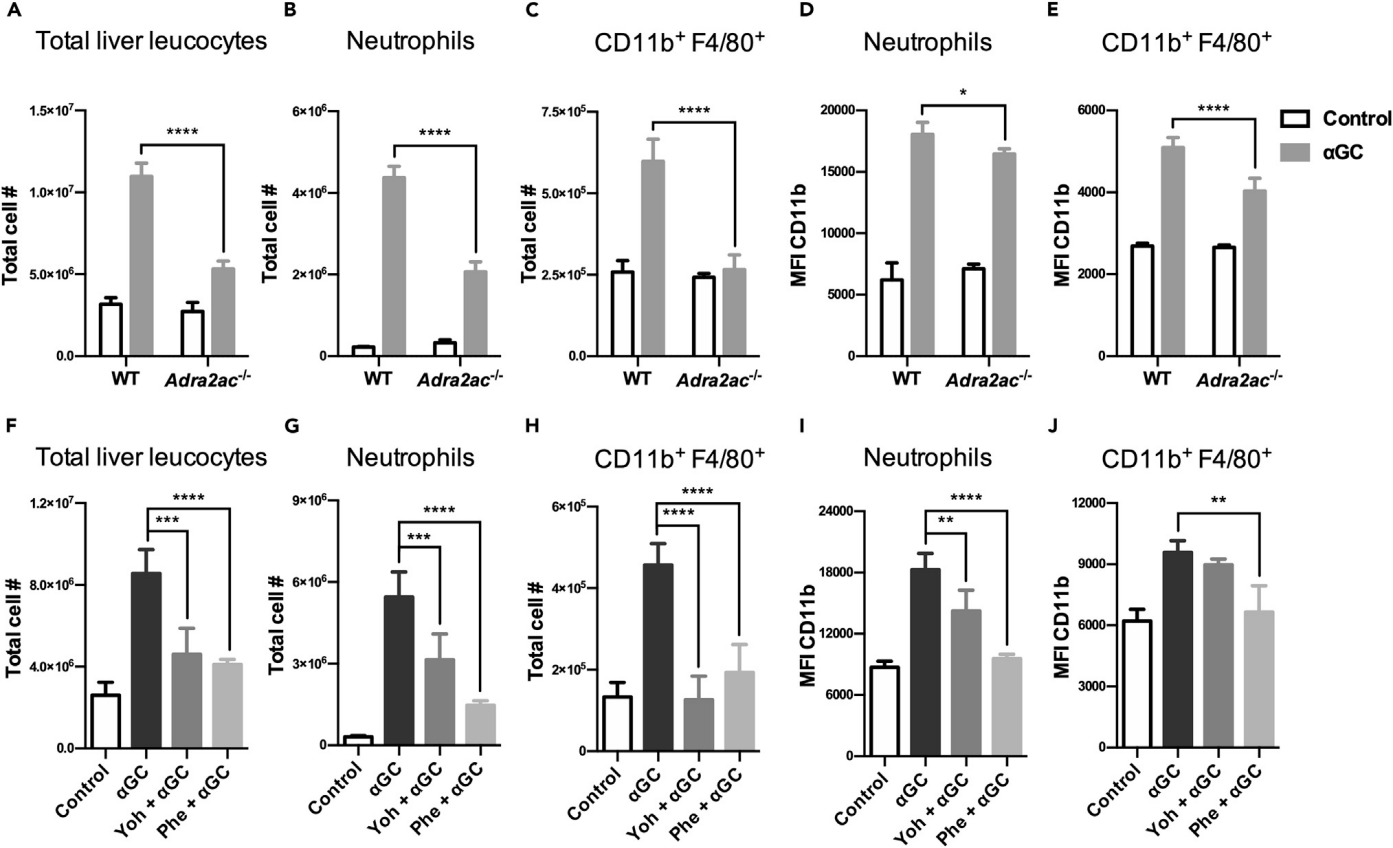
Adrenergic signaling regulates CD11b expression in myeloid cells and controls migration into the liver after αGC treatment (A–E) C57BL/6J WT and *Adra2ac*^−/−^ mice were treated with αGC (2μg/animal), and liver leukocytes were analyzed by flow cytometry after 3 h. Graphs represent the total number of liver leukocytes (CD45^+^) (A), neutrophils (CD11b^+^Ly6G^+^) (B) and CD11b^+^F4/80^+^ cells (C), as well as the CD11b MFI in neutrophils (D) and CD11b^+^F4/80^+^ cells (E). (F–J) C57BL6/J WT animals were pre-treated with Yoh (5 mg/kg) or Phe (10 mg/kg) and then treated with αGC (2μg/animal). Liver leukocytes were analyzed by flow cytometry after 3 h, and graphs represent the total number of liver leukocytes (CD45^+^) (F), neutrophils (CD11b^+^Ly6G^+^) (G) and CD11b^+^F4/80^+^ cells (H), as well as the CD11b MFI in neutrophils (I) and CD11b^+^F4/80^+^ cells (J). Data represent the mean ± SD from a single set of two independent experiments (n = 5). ∗p < 0.05, ∗∗p < 0.01, ∗∗∗p < 0.001, ∗∗∗∗p < 0.0001.

To determine the influence of adrenergic signaling on the influx of inflammatory cells into the liver after iNKT cell activation, we first analyzed the profile of neutrophils and inflammatory macrophage-like cells (CD11b^+^F4/80^+^) in the liver of *Adra2ac*^−/−^ mice. Although αGC administration induced the influx of inflammatory leukocytes into the hepatic parenchyma in both the WT and *Adra2ac*^−/−^ mice, the latter animals exhibited fewer infiltrating cells (Figure 8A). As a result, the total number of neutrophils and CD11b^+^F4/80^+^ was lower in the *Adra2ac*^−/−^ mice (Figures 8B and 8C). Flow cytometric analysis also revealed a reduction in the expression of CD11b in both cellular populations in these animals, indicating a lower pro-inflammatory profile compared to the WT group (Figures 8D and 8E).

Therefore, these data support the notion that although adrenergic signaling does not influence the iNKT cells response to αGC, *Adra2ac*^−/−^ mice exhibit a reduced inflammatory reaction due to adrenergic signaling on myeloid-derived cells. Indeed, pharmacological modulation of the adrenergic response by Yoh or Phe treatment replicated the same inflammatory profile observed in *Adra2ac*^−/−^ mice, as both treatments reduced the influx of inflammatory cells (Figures 8F–8H). Notably, Phe’s influence on myeloid-derived cells appears more pronounced than that of Yoh, suggesting a more suppressive effect for α_1_AR adrenergic stimulation (Figures 8I and 8J).

## Discussion

Alterations in neuro-endocrine-immune homeostasis promoted by the activation of the sympathetic nervous system are linked to susceptibility to tumor development and resistance to chemo/radiotherapies.^36^^,^^37^ As αGC-based immunotherapy is highlighted as an alternative approach to overcome tumor resistance to conventional therapies, we aimed to determine the impact of adrenergic signaling on the antimetastatic activity of iNKT cells. We observed that a combination of SNS stimulation and iNKT cell activation could promote tumor elimination without the deleterious liver damage associated with αGC therapy.

Norepinephrine signaling through the β_2_AR during the *ex vivo* stimulation of conventional naive CD4^+^ T lymphocytes impairs IFN-γ production, leading to a skewing toward the Th2 profile.^19^^,^^21^ Therefore, considering that αGC’s ability to control tumor growth depends on the production of IFN-γ by iNKT cells, which express α and βARs, our first question was to determine whether adrenergic signaling impairs their activation toward a type 1 profile.^24^^,^^38^ In this context, *in vitro* treatment with NE did not inhibit the production of IFN-γ or TNF-α by splenic iNKT cells upon cognate antigen stimulation. Due to the existence of tissue-specific iNKT cell profiles with their particular cytokine responses and the fact that the hepatic-resident IFN-γ-producing iNKT cells are the most effective mediators of *in vivo* antitumor activity of αGC, we took advantage of the *Adra2ac*^−/−^ mouse strain to determine the influence of NE signaling on liver-resident iNKT cells.^38^^,^^39^^,^^40^^,^^41^ In line with the concept that iNKT cells coordinate various immune responses, both WT and the *Adra2ac*^−/−^ mouse strains exhibited a substantial and similar increase in systemic levels of several immunomodulatory molecules following αGC administration.^42^ These results thus supported the idea that iNKT cells, unlike other immune cells, are refractory to the suppressive effect of adrenergic signaling.

An important aspect of the iNKT cell biology is that their rapid activation by αGC stimulation results in a dual-edged immune reaction. While it potentiates host resistance against pathogens or cancer through type-1 immunity, it also triggers a cascade of inflammatory events leading to liver injury. Several experimental studies have implicated TNF-α-producing iNKT cells in hepatocyte damage, characterized by elevated systemic ALT and AST levels and the emergence of necrotic areas in liver parenchyma.^27^^,^^43^^,^^44^

In contrast to what was observed in WT mice, αGC administration in *Adra2ac*^−/−^ animals did not result in liver dysfunction despite the cytokine storm, the presence of hepatic TNF-α-producing iNKT cells, *in situ*, and a significant influx of inflammatory cells into liver parenchyma. Therefore, these data suggested that instead of suppressing the αGC-induced immune response, adrenergic signaling promotes hepatocyte protection against iNKT-mediated cytotoxicity, raising the question of whether this phenomenon could be extended to malignant cells. To answer this question, we decided to determine the impact of adrenergic signaling on the antimetastatic capacity of αGC-activated iNKT cells.

While the sympathetic nervous system’s hyperactivity rendered *Adra2ac*^−/−^ mice more susceptible to lung metastasis, αGC treatment overcame the pro-metastatic effect of the adrenergic signaling, promoting melanoma control with the same efficiency observed in WT mice. Despite the antitumor activity, we did not detect any signal of liver injury in the αGC-treated *Adra2ac*^−/−^ mice, in contrast to WT mice injected with αGC. Therefore, these results underscored adrenergic signaling as a target for modulating liver lesions during iNKT-based immunotherapy.

Considering adrenergic signaling as a target to improve αGC immunotherapy, we hypothesized that the punctual stimulation of NE release could maintain liver physiology despite iNKT cell activation. To mimic the *Adra2ac*^−/−^ mice phenotype, we pre-treated C57BL/6J WT mice with Yoh, an α_2_AR antagonist.^29^ Alongside the positive response to the antimetastatic effect of αGC treatment, the liver physiology was preserved in tumor-bearing Yoh-pre-treated mice, further supporting the idea that adrenergic signaling protects hepatocytes from iNKT-mediated injury. Notably, the hepatoprotective effect provided by Yoh pre-treatment extended to PBS44, an αGC analog that shares its antimetastatic ability, serving as another potential immunotherapeutic agent.^45^ These results thus corroborated the idea that stimulating sympathetic fibers is an alternative to improving the outcomes of iNKT-based immunotherapy.

Finally, we directed our focus toward the signaling pathways driving adrenergic-mediated hepatocyte protection. Under steady-state conditions, the most expressed adrenergic receptor in hepatocytes is the α_1B_AR (*Adra1b*>*Adra2b*>*Adrb*3>*Adra1a*), which could account for the maintenance of liver physiology/structure in the Adra2ac^−/−^ mice.^31^ Despite a study showing the induction of β_2_AR following partial hepatectomy (PHx), our findings did not associate β_2_AR signaling with the protection against iNKT-induced hepatotoxicity.^33^ Using the *Adra2ac*^−/−^*Adrb2*^−/−^ mice, we demonstrated that even with β_2_AR deficiency, the liver injury induced by αGC administration remained under the control of the adrenergic signaling promoted by the absence of *Adra2ac*, indicating that β_2_AR is dispensable for hepatocyte protection against immune-mediated injury. Although our approach cannot entirely exclude the participation of the other members of βAR family, no evidence supports a role for β_1_ or β_3_AR in injury-induced hepatocyte survival/proliferation.^33^ Regarding the potential implication of α_2B_AR, a recent study demonstrated that in the case of PHx, the regenerative effect of an α_2_AR agonist is due to macrophage differentiation toward an anti-inflammatory profile and not from direct action on hepatocytes.^46^ Since we did not find any indication of alteration in the pro/anti-inflammatory cytokines ratio due to the adrenergic signaling, we supposed that this was not the principal mechanism maintaining liver physiology after iNKT cell activation. Consequently, we opted to pre-treat mice with Phe, a selective α_1_AR agonist, before αGC administration.^47^ This approach inhibited the development of liver injury induced by iNKT cell activation without altering the production of pro-inflammatory cytokines induced by αGC.

Previous studies have demonstrated that liver injury due to αGC-induced iNKT cell activation depends on the FasL/Fas pathway and TNF-α production.^13^^,^^27^ Although TNF-α alone cannot induce hepatocyte apoptosis, it does stimulate Fas expression on their surface, rendering them susceptible to FasL-induced cell death.^48^ Considering that the adrenergic signaling does not impair TNF-α production, our initial hypothesis was that it might protect hepatocytes from apoptosis by inhibiting the FasL/Fas pathway. To test this assumption, we stimulated AML12 cells with NE or Phe and then incubated them with TNF-α or FasL.

However, both stimuli failed to inhibit TNF-α-induced Fas expression on the AML12 cell surface or prevent their death via the FasL/Fas pathway. This suggested that adrenergic signaling does not have a direct impact on hepatocytes. To determine the influence of adrenergic signaling on iNKT-mediated hepatocyte apoptosis, we then co-cultured αGC-pulsed AML12 cells with hepatic leukocytes pre-incubated with NE or Phe. Corroborating the notion that iNKT cells are resistant to the suppressive effect of adrenergic signaling, αGC-pulsed AML12 cells underwent apoptosis upon contact with hepatic leukocytes. Therefore, these results demonstrated that adrenergic signaling does not directly affect hepatocytes or iNKT-induced apoptosis.

In addition to the implication of the TNF-α/FasL axis in hepatic apoptosis, iNKT cell activation also induces neutrophil accumulation in the liver parenchyma as early as 3 h, accompanied by an increase in caspase3.^35^ Neutrophil depletion through anti-Ly6G administration inhibited liver injury, in contrast to STAT1^−/−^ deficiency, which increased infiltrating neutrophils and hepatic damage despite lower levels of Fas expression compared to the WT mice.^35^ Therefore, apart from TNF-α/FasL-mediated hepatocyte apoptosis, early neutrophil infiltration contributes to liver injury.

In this context, we observed that *in vitro* adrenergic stimulation impaired the acquisition of a more inflammatory/invasive profile by neutrophils (Ly6G^+^CD11b^high^) treated with NE or Phe before incubation with αGC-pulsed AML12 cells.^49^^,^^50^ In line with this, WT animals exhibited higher hepatic leukocyte counts after αGC administration than the *Adra2*ac^−/−^ mice. Furthermore, in the latter, the infiltrating neutrophils and myeloid-derived CD11b^+^F4/80^+^ mice expressed lower levels of CD11b on their surface, indicating a lesser inflammatory/invasive profile due to adrenergic signaling. Pharmacological stimulation of adrenergic signaling in the WT mice, by Yoh or Phe, replicated the lower inflammatory profile after αGC administration. The hypothesis that NE and Phe modulate the inflammatory profile aligns with previous studies showing the influence of adrenergic receptors on innate immunity.^20^

In short, αGC administration triggers two hepatotoxic self-limiting pathways. One pathway depends on iNKT-mediated activation of the TNF-α/FasL axis, while another is promoted by the influx of myeloid-derived inflammatory cells, leading to more extensive hepatic damage as reflected by the increase in serum ALT/AST levels. In this context, although adrenergic signaling fails to modulate the cognate activation of iNKT cells and the TNF-α/FasL-induced hepatocyte death, it does restrain the inflammatory response of the myeloid cells and, consequently, mitigates liver injury.

Although further experiments are required to elucidate the mechanisms underlying the protective effect observed upon sympathetic stimulation, it is plausible to assume that signaling through the α_1_AR is the primary pathway that prevents iNKT-induced liver damage.

In conclusion, our results demonstrate that modulating adrenergic signaling during αGC immunotherapy preserves liver physiology without compromising the control of tumor growth. Beyond enhancing the αGC therapeutic outcomes, this approach can also be extended to other iNKT agonists and offers new perspectives in managing immune-mediated liver diseases.

### Limitations of the study

While we have demonstrated that iNKT cells are refractory to adrenergic signaling, which in turn modulates αGC-induced hepatocyte apoptosis, further experiments are necessary to elucidate the mechanisms underlying these phenomena.

## STAR★Methods

### Key resources table

**Table:** 

REAGENT or RESOURCE	SOURCE	IDENTIFIER
**Antibodies**		

Anti-mouse CD3e Monoclonal Antibody, PE	eBioscience™	Clone: 145-2C11, Catalog # 12-0031-85; RRID: AB_465498
Anti-mouse NK1.1 Monoclonal Antibody, FITC	eBioscience™	Clone: PK136, Catalog # 11-5941-82; RRID: AB_465318
Anti-mouse IFN gamma Monoclonal Antibody, APC-eFluor™ 780	eBioscience™	Clone: XMG1.2, Catalog # 47-7311-82; RRID: AB_2688060
Anti-mouse TNF alpha Monoclonal Antibody, PerCP-eFluor™ 710	eBioscience™	Clone: MP6-XT22, Catalog # 46-7321-82; RRID: AB_1834445
Anti-mouse CD45 Monoclonal Antibody, PE-Cyanine7	eBioscience™	Clone: 30-F11, Catalog # 25-0451-82; RRID: AB_2734986
Anti-mouse CD95 (APO-1/Fas) Monoclonal Antibody, PE	eBioscience™	Clone: 15A7, Catalog # 12-0951-81; RRID: AB_465788
Anti-mouse CD11b Monoclonal Antibody, PE	eBioscience™	Clone: M1/70, Catalog # 12-0112-82; RRID: AB_2734869
Anti-mouse Ly-6G Monoclonal Antibody, APC-eFluor™ 780	eBioscience™	Clone: 1A8, Catalog # 47-9668-82; RRID: AB_2802291
Anti-mouse F4/80 Monoclonal Antibody, eFluor™ 450	eBioscience™	Clone: BM8, Catalog # 48-4801-82; RRID: AB_1548747
Anti-mouse CD16/CD32 Monoclonal Antibody (FC block)	eBioscience™	Clone: 93, Catalog # 14-0161-82; RRID: AB_467133
Anti-mouse CD3 Monoclonal Antibody, Functional Grade	eBioscience™	Clone: 17A2, Catalog # 16-0032-82; RRID: AB_468851
Anti-mouse CD28 Monoclonal Antibody, Functional Grade	eBioscience™	Clone: 37.51, Catalog # 16-0281-82; RRID: AB_468921

**Chemicals, peptides, and recombinant proteins**		

Human TNF-α recombinant protein	Adipogen®	Catalog #: AG-40B-0006-C010
Human FasL recombinant protein	Adipogen®	Catalog #: AG-40B-0001-C010
TNF Ligands Enhancer	Adipogen®	Catalog #: AG-35B-0001-C050
KRN 7000 (αGalCer)	Cayman Chemical	Catalog #: 11208
Brefeldin A Solution	eBioscience™	Catalog # 00-4506-51
Monensin Solution	eBioscience™	Catalog # 00-4505-51
murineCD1d/PBS-57	NIH Tetramer Core Facility	N/A
Phenylephrine hydrochloride	Sigma-Aldrich®	Catalog #: P6126
Yohimbine hydrochloride	Sigma-Aldrich®	Catalog #: Y3125
Norepinephrine bitartrate salt monohydrate	Sigma-Aldrich®	Catalog #: A9512
DMEM, high glucose	Gibco™	Catalog #: 11965118
RPMI 1640 Medium	Gibco™	Catalog #: 11875093
Fetal Bovine Serum	Gibco™	Catalog #: 10437028
Penicillin-Streptomycin	Gibco™	Catalog #: 15140122
DMEM/F-12	Gibco™	Catalog #: 11320033
HEPES	Gibco™	Catalog #: 15630080
Sodium Pyruvate	Gibco™	Catalog #: 11360070
Insulin-Transferrin-Selenium (ITS -G)	Gibco™	Catalog #: 41400045
L-Glutamine	Gibco™	Catalog #: A2916801
Dexamethasone	Gibco™	Catalog #: A13449
MEM Non-Essential Amino Acids Solution	Gibco™	Catalog #: 11140050
MEM Vitamin Solution	Gibco™	Catalog #: 11120052
PBS44	Paul B. Savage. BYU Chemistry and Biochemistry. Provo, USA	N/A

**Critical commercial assays**		

IFN gamma Mouse Uncoated ELISA Kit	Invitrogen™	Catalog # 88-7314-88
IL-4 Mouse Uncoated ELISA Kit	eBioscience™	Catalog #88-7044-88
IL-17AF (heterodimer) Mouse Uncoated ELISA Kit	Invitrogen™	Catalog # 88-8711-88
Cytometric Bead Array (CBA) Mouse Inflammation Kit	BD™	Catalog #: 552364
AST Kinetic UV	Bioclin	Catalog #: K048
ALT Kinetic UV	Bioclin	Catalog #: K049
Intracellular Fixation & Permeabilization Buffer Set	eBioscience™	Catalog # 88-8824-00
LIVE/DEAD™ Fixable Aqua Dead Cell Stain Kit	Invitrogen™	Catalog #L34957

**Experimental models: Cell lines**		

AML12 cell line	BCRJ	BCRJ code: 0354
B16F10 cell line	BCRJ	BCRJ code: 0046

**Experimental models: Organisms/strains**		

Mouse: C57BL6/J	CEDEME/UNIFESP	RRID:IMSR_JAX:000664
Mouse: *Adra2ac*^−/−^ B6 background	CEDEME/UNIFESP	N/A
Mouse: *Adrb2*^−/−^ B6 background	CEDEME/UNIFESP	N/A
Mouse: *Adra2ac*^−/−^*Adrb2*^−/−^ B6 background	CEDEME/UNIFESP	N/A

**Software and algorithms**		

FlowJo™ v10 Software	BD Biosciences	N/A
GraphPad Prism 7 Software	GraphPad	N/A

### Resource availability

#### Lead contact

Further information and requests for resources and reagents should be directed to and will be fulfilled by the Lead Contact, Alexandre Castro Keller (ackeller@unifesp.br).

#### Materials availability

This study did not generate new unique reagents.

### Experimental model and study participant details

#### Mice

Male C57BL/6J wild type (WT), *Adra2ac*^−/−^, *Adrb2*^−/−^, and *Adra2ac*^−/−^*b2*^−/−^ mice, aged 8–10 weeks, were obtained from CEDEME (Centro de Desenvolvimento de Modelos Experimentais para Medicina e Biologia).^51^ All animals were housed in a specific pathogen-free environment with access to filtered water and standard solid food *ad libitum*. All animal procedures were conducted in accordance with Federal Law 11.794 (2008), The ARRIVE guidelines, and the Guide for the Care and Use of Laboratory Animals of the Brazilian National Council of Animal Experimentation (CONCEA). These procedures were approved by the local ethical committee (Comissão de Ética no Uso de Animais - CEUA) (9831160519).

#### Spleen cells culture

Splenic and liver murine cells were cultured in RMPI 1640 medium supplemented with 10% FBS, 100μM sodium pyruvate, 2μM L-glutamine, 10μM HEPES, 100U/mL of Penicillin/Streptomycin, non-essential amino acids (NEEA), and Vitamins at 37°C with 5% of CO2.

#### B16F10 culture

B16F10 cells, derived from a murine melanoma cell line, were cultured in Dulbecco’s Modified Eagle Medium (DMEM) supplemented with 10% Fetal Bovine Serum (FBS) and 100U/mL of Penicillin/Streptomycin at 37°C with 5% of CO2.

#### AML12 culture

AML12 cells, derived from a murine hepatocyte cell line, were cultured in DMEM/F12 medium supplemented with 10% FBS, 1mM sodium pyruvate, 15mM HEPES, 2.5mM L-glutamine, 100U/ml penicillin, 10 μg/ml streptomycin, 10 μg/ml insulin, 5.5 mg/ml transferrin and 5 ng/ml selenium at 37°C with 5% of CO2.

### Method details

#### *In vivo* treatment with α-galactosylceramide

Animals were treated with 2μg of αGC via intraperitoneal injection in 200μL of 0.9% saline. The control group received saline as a vehicle solution. To analyze the iNKT response to αGC *ex vivo* through intracellular staining, spleen and liver samples were collected 90 min after αGC administration, and cells were cultured for 2 hours in the presence of brefeldin A and monensin.^52^ The experiments with Yoh or Phe followed the same protocol, but animals were respectively treated with the pharmacological α_2_AR antagonist (5 mg/kg) or the α_1_AR agonist (10 mg/kg) via IP route in 0.9% saline, 30 minutes before αGC.

#### Spleen cells activation *in vitro*

For *in vitro* activation assay, splenic cells were incubated with or without NE (1μM) for 30 minutes, stimulated with anti-CD3/CD28 (2 μg/ml) or mCD1d/PBS57 monomer (1 μg/ml) and anti-CD28(2 μg/ml) for 1 hour, followed by additional 2 hours in the presence of brefeldin A and monensin.

#### AML12 *in vitro* assay

For the evaluation of CD95 expression, cells were incubated for 2 hours with or without NE or Phe (10μM) and then treated with recombinant TNF-α (20 ng/ml) for 16 hours. To analyze cell death, AML12 cells were incubated for 2 hours with or without NE or Phe (10μM), followed by treatment with recombinant TNF-α (20 ng/ml) for 4 hours, and then treated with recombinant FasL (100 ng/ml) in the presence of TNF ligand enhancer (1 μg/ml) for an additional 12 hours. Cytotoxicity was assessed by measuring the release of LDH in the supernatant, using triton X-100 (1%) as a positive control for cell death. To evaluate αGC-induced cell death, AML12 cells were pulsed with αGC (250 ng/ml) overnight, followed by incubation with or without NE or Phe (10μM) for 2 hours. Subsequently, these cells were co-cultured with liver leucocytes, pre-treated with NE or Phe, for 4 h.

#### Flow cytometry analysis

For all experiments, cells were initially stained with Aqua Fluorescent Viability Dye in the presence of Fc-block (anti-CD16/32). Intracellular cytokine evaluation involved staining cells with surface markers against CD3, NK1.1, and the invariant TCR (mCD1d/PBS57 tetramer), followed by fixation, permeabilization, and intracellular staining to quantify IFN-γ and TNF-α-producing iNKT cells (CD3^+^mCD1d/PBS57^+^) and conventional T cells (CD3^+^mCD1d/PBS57^−^NK1.1^-^). Gate strategies are represented in Figure S1.

In AML12 pure cultures, staining was performed with surface marker against CD95. In co-cultures of AML12 and liver leucocytes, cells were detached with trypsin 0.25% and stained with surface markers against CD45, CD11b, and Ly6G, followed by AnnexinV staining. AnnexinV analysis was carried out in the CD45^−^ compartment, and CD11b expression was measured in neutrophils (CD45^+^CD11b^+^Ly6G^+^).

For analysis of the early infiltrating content, the liver was collected 3 hours after αGC treatment and stained with markers against CD11b, F4/80 and Ly6G. The cellular content and CD11b expression in neutrophils (CD11b^+^ Ly6G^+^) and CD11b^+^F4/80^+^ cells were assessed.

#### Quantification of *in vivo* cytokine production

Blood was collected through submandibular puncture of the facial vein 4 hours after αGC administration to assess systemic levels of pro-inflammatory molecules using CBA or ELISA assays. In certain experiments, the systemic IFN-γ levels were measured 16 hours after αGC injection.

#### Analysis of liver injury

Liver injury was assessed by measuring the systemic levels of the hepatic enzymes alanine aminotransferase (ALT) and aspartate aminotransferase (AST), in serum samples collected 16 hours after αGC administration, using commercial kits (Bioclin, BR). In some experiments, organs were harvest, fixed, and paraffin-embedded, and tissue slices with a thickness of 5μm were stained with hematoxylin/eosin solutions for histological analysis. Briefly, digital images were captured using a 4x and 10x objective on a light microscope (Olympus, PA) equipped with a digital camera (Moticam 500, Motic, CA) for histopathologic evaluation. Samples were coded and subsequently evaluated by a trained pathologist unaware of the treatment modalities, scoring them according to observed alterations, such as inflammatory infiltrates and necrotic areas,. The scoring system ranged from zero (no alterations) to 3 (severe alterations). The results represent the mean score obtained for each parameter (0–3).

#### B16F10 metastatic tumor model

Animals were grafted with 2x10^5^ B16F10 melanoma cells through the tail vein in 100μL of 0.9% saline. They were subsequently treated with 2μg of αGC via intraperitoneal (IP) injection. In experiments involving Yoh, the same protocol was followed, with animals receiving the pharmacological α_2_AR antagonist (5 mg/kg via IP route in 0.9% saline) 30 minutes prior to engraftment. Euthanasia was realized 15 days after grafting, and tumor burden was assessed by counting the pulmonary tumor nodules. In some experiments animals received 2μg of PBS44 via intraperitoneal (IP) injection.

### Quantification and statistical analysis

Statistical comparisons were conducted using ANOVA followed by Turkey’s multiple comparison test for comparisons involving three or more groups. For comparisons between two isolated groups, the Mann-Whitney test was employed. Statistical details of the experiments can be found in the figure legends. All analyses were performed using GraphPad Prism 7 software (GraphPad Software Inc., EUA). p values < 0.05 were considered statistically significant.

## Data Availability

Data reported in this paper will be shared by the lead contact upon request. This paper does not report original code. Any additional information required to reanalyze the data reported in this paper is available from the lead contact upon request.
