# Supplementary material for: Targeting adrenergic receptors to mitigate invariant natural killer T cells-induced acute liver injury
Source: iScience. 2023 Sep 16;26(10):107947. doi: 10.1016/j.isci.2023.107947 (PMC10568435; doi:10.1016/j.isci.2023.107947)
Supplement: Document S1. Figures S1–S3 [file mmc1.pdf]

iScience, Volume *26*

# Supplemental information

# Targeting adrenergic receptors to mitigate invariant natural killer T cells-induced acute liver injury

**Michelangelo Bauwelz Gonzatti, Beatriz Marton Freire, Maísa Mota Antunes, Gustavo Batista de Menezes, Jhimmy Talbot, Jean Pierre Schatzmann Peron, Alexandre Salgado Basso, and Alexandre Castro Keller**

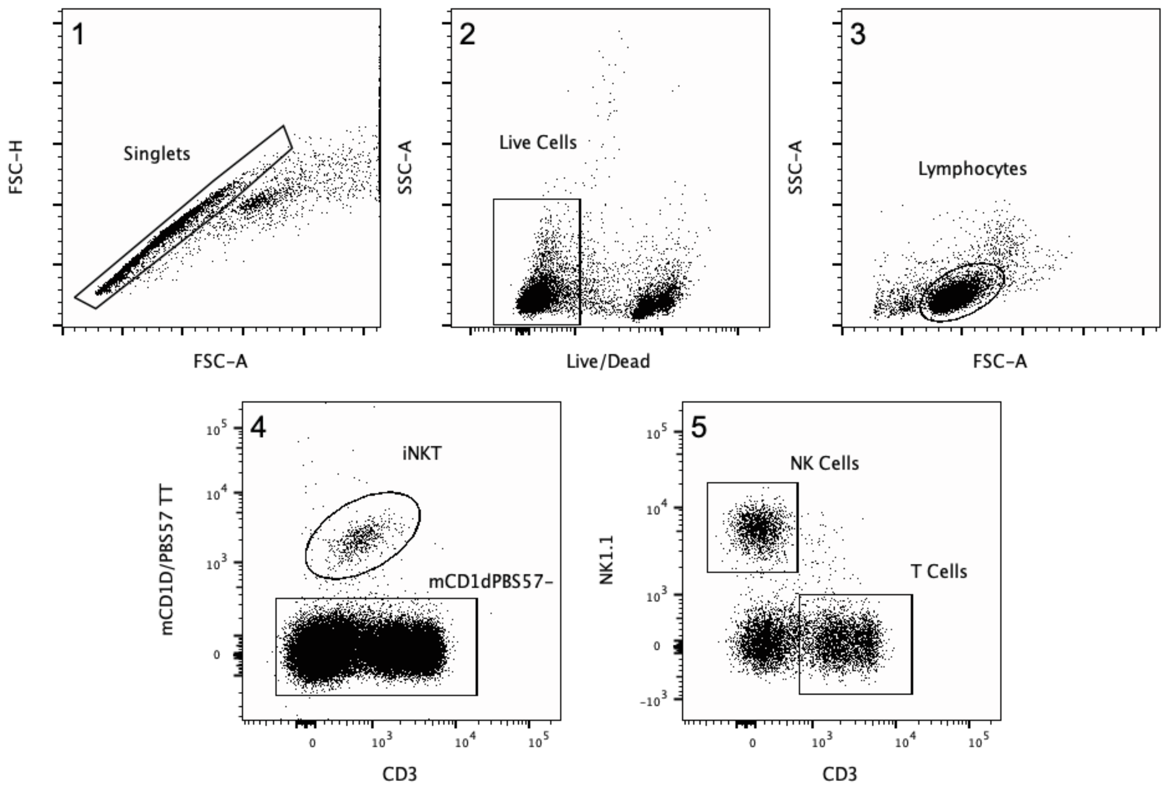


**Figure S1. Representative Flow cytometric strategies for iNKT cells and conventional T lymphocytes analysis, related to Figures 1 and 2.** 1- Removal of doublets; 2- Live cells selection; 3- Lymphoid population selection using SSC-A x FSC-A; 4- iNKT cells analysis as $CD3^{+}$ mCD1d/$PBS57^{+}$; 5- Conventional T lymphocytes analysis as Tetramer $CD3^{+}$ mCD1d/$PBS57^{-}$ $NK1.1^{-}$.

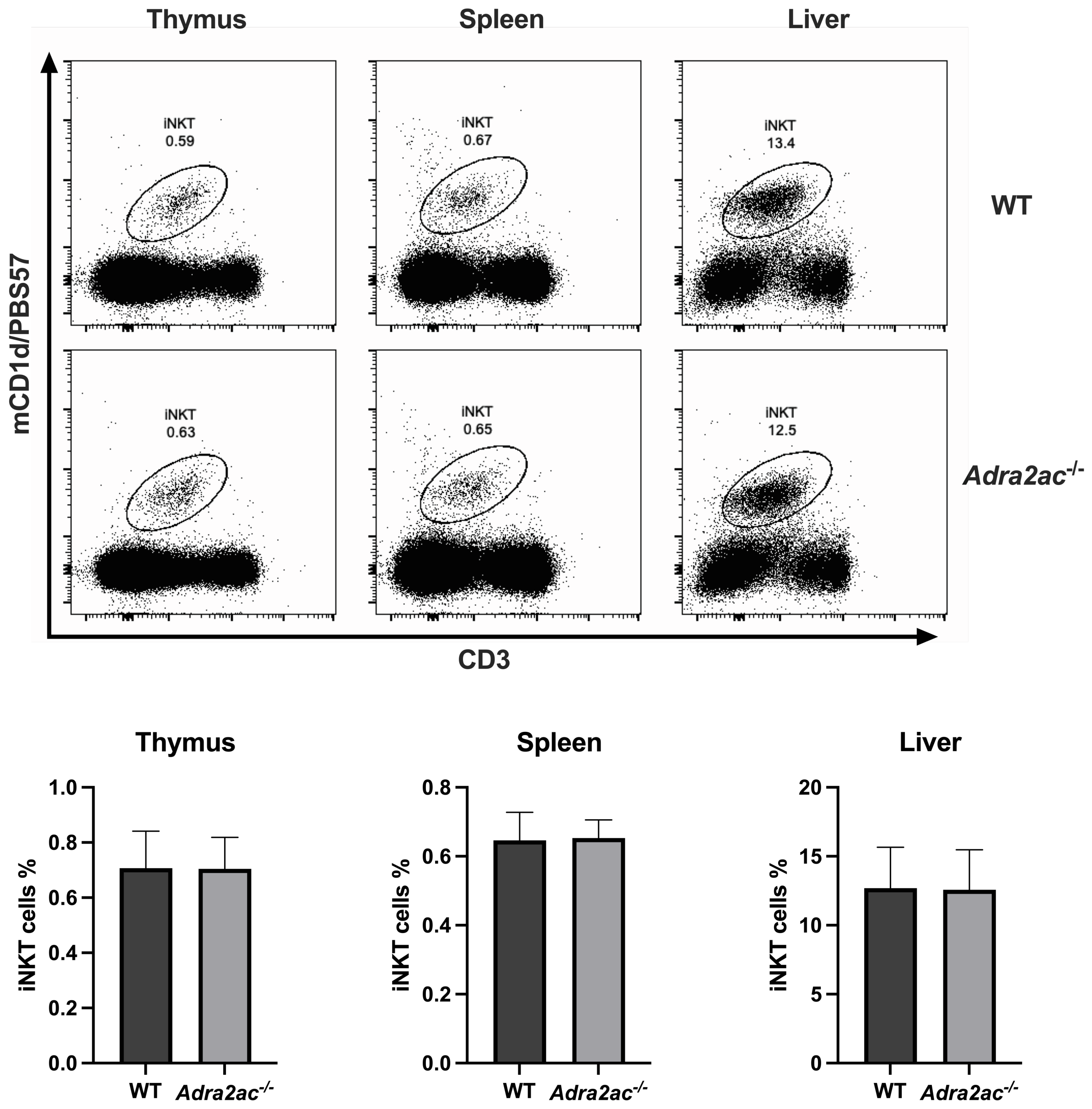


**Figure S2. Deletion of $\alpha_{2A}$ and $\alpha_{2C}$AR does not affect the distribution of iNKT cells, related to Figure 2.** Cells from thymus, spleen, and liver from C57BL/6J WT and *Adra2ac* mice were stained with surface markers for evaluation of iNKT cells ($CD45^+$, $CD3^+$, mCD1d/$PBS57^+$). Data represents mean ± SD of iNKT percentage among total $CD45^+$ cells after doublets and dead cells exclusion. Data represent the mean ± SD from a single set of two independent experiments (n=5).

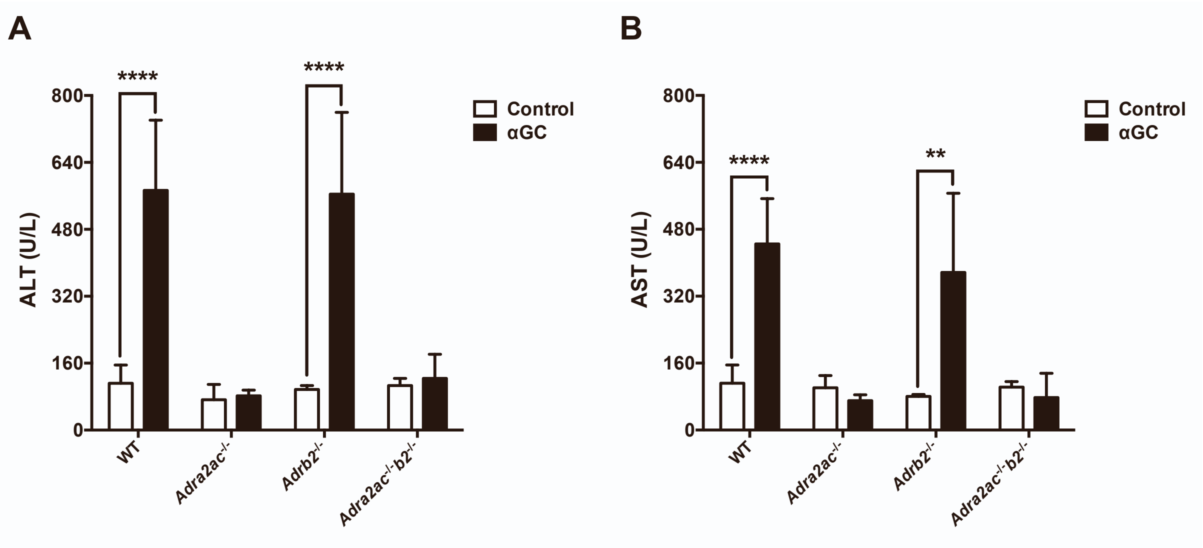


**Figure S3. The $\beta_2$AR is dispensable for hepatoprotective effect provided by adrenergic signaling, related to Figure 3.** C57BL/6J WT, *Adra2ac*$^{-/-}$, *Adrb2*$^{-/-}$, *Adra2ac*$^{-/-}$ *b2*$^{-/-}$ and mice were treated with αGC. ALT (A) and AST (B) serum levels were evaluated after 16 hours. Mean ± SD of 1 experiment (n=4-8). **** $p<0.0001$; ** $p<0.01$.
